# PReS-FINAL-2013: Sodium thiosulfate for the treatment of Juvenile Dermatomyositis complicated by calcinosis

**DOI:** 10.1186/1546-0096-11-S2-P26

**Published:** 2013-12-05

**Authors:** I Pagnini, G Simonini, T Giani, L Cantarini, R Cimaz

**Affiliations:** 1 AOU Meyer-Rheumatology Department, Florence, Italy; 2 Rheumatology Unit, Siena, Italy

## Introduction

Juvenile Dermatomyositis (JDM) is an inflammatory myopathy with a predilection for proximal muscles and skin. Current treatment of JDM includes early aggressive use of corticosteroids coupled with immunosuppressive drugs such as methotrexate, cyclosporine and intravenous immune globulins (IVIG). Delay to diagnosis and inadequate treatment increase the risk of developing calcinosis. However, once estabilished, this complication is difficult to treat.

## Objectives

We describe a case of JDM with severe skin manifestations including ulcerations and diffuse calcinosis, poorly responsive to conventional therapy.

## Methods

**Case report. **A caucasian boy presented at the age of 3 years and 10 months with malaise, fatigue, arthralgia, heliotrope rash, Gottron's papules on PIP joints, elbows and knees, shawl sign, and periungueal telangectasia. There was weakness of proximal muscles, elevation of aldolase (11.0 U/L, normal < 7.3 U/L), normal serum lactate dehydrogenase (LDH) and creatine kinase (CK), and negative anti-nuclear antibody. Magnetic resonance imaging (MRI) demonstrated diffuse muscle edema of proximal muscles and electromyography showed a myopathic pattern. Despite treatment with steroids pulses, followed by daily oral steroids and hydroxycloroquine, his illness was complicated by skin ulcerations of the upper and lower extremities and widespread calcinosis. X-ray of extremities showed superficial diffuse nodules and plaques. The patient still had elevation of aldolase (19.3 U/L) and a Childhood Myositis Assessment Scale (CMAS) score of 35 out of 52, with a Manual Muscle Testing (MMT8) score of 50 out 80. So a treatment with Methotrexate was added, but his areas of calcinosis and ulcerations continued to spread, so alendronate was introduced (two years from disease onset), but without benefit. On the basis of a published report *(Arabshahi B, et al. J Pediatr 2012) *treatment with topical sodium thiosulfate was initiated. Thiosulphate was used initially at 3% concentration, and subsequently increased to 10% concentration, applied to calcification of upper and lower extremities daily under occlusive dressing.

## Results

After 9 months of therapy, a significant improvement of calcinosis and ulcerations was noted, together with lack of progression. Photos, with the family consent, were taken before and after treatment.

## Conclusion

Treatment of calcinosis in JDM is very difficult. Several agents have been used, such as calcium channel blockers, probenecid, colchicine, tumor necrosis factors inhibitors, bisphosphonates, and intra-lesional corticosteroids. None of these however has shown to be consistently effective and no controlled trial exist. Sodium thiosulfate is a potent antioxidant and vasodilator that also chelates and dissolves calcium deposits. Topical use has been described for ulcerations associated with lupus calcinosis and uremic calciphylaxis. However, the use of this agent for treatment of calcinosis associated to dermatomyositis has been reported only once in literature.

We hypothesize that sodium thiosulfate may have a role in stabilizing or improving calcinosis, and in diminishing pain and promoting revascularization of cutaneous ulcerations. A controlled study in order to determinate the safety and efficacy of this treatment in JDM is currently planned.

## Disclosure of interest

None declared.

